# A framework for cross-cultural development and implementation of complex interventions to improve palliative care in nursing homes: the PACE steps to success programme

**DOI:** 10.1186/s12913-019-4587-y

**Published:** 2019-10-24

**Authors:** Jo Hockley, Katherine Froggatt, Lieve Van den Block, Bregje Onwuteaka-Philipsen, Marika Kylänen, Katarzyna Szczerbińska, Giovanni Gambassi, Sophie Pautex, Sheila Alison Payne, on behalf of PACE, Lieve Van den Block, Borja Arrue, Ilona Baranska, Luc Deliens, Yvonne Engels, Harriet Finne-Soveri, Katherine Froggatt, Giovanni Gambassi, Viola Kijowska, Maud ten Koppel, Marika Kylanen, Federica Mammarella, Tinne Smets, Bregje Onwuteaka-Philipsen, Mariska Oosterveld-Vlug, Roeline Pasman, Sheila Payne, Ruth Piers, Lara Pivodic, Jenny van der Steen, Katarzyna Szczerbińska, Nele Van Den Noortgate, Hein van Hout, Anne Wichmann, Myrra Vernooij-Dassen

**Affiliations:** 1 International Observatory on End of Life Care, Faculty of Health and Medicine, Lancaster University, Lancaster, LA1 4YG UK; 2 Usher Institute, University of Edinburgh, Edinburgh, EH8 9AG UK; 3 Department of Family Medicine and Chronic Care, End-of- Life Care Research Group, Vrije Universiteit Brussel (VUB) and Ghent University, Brussels, Belgium; 4 Amsterdam UMC, Vrije Universiteit Amsterdam, Department of Public and Occupational Health, Amsterdam Public Health research institute, Amsterdam, the Netherlands; 5 National Institute for Health and Welfare, Helsinki, Finland; 6 Unit for Research on Aging Society, Department of Sociology, Chair of Epidemiology and Preventive Medicine, Faculty of Medicine, Jagiellonian University Medical College, Kraków, Poland; 7 Fondazione Policlinico Universitario A. Gemelli IRCCS, Rome, Italy; 8 Universita’ Catholica del Sacro Cuore, Rome, Italy; 9 Division of Palliative Medicine, University Hospital Geneva and University of Geneva, Geneva, Switzerland

**Keywords:** Cross-cultural adaptation, Development, Implementation, Palliative care, End-of-life care, Long-term care facilities, Nursing home, Intervention

## Abstract

**Background:**

The PACE Steps to Success programme is a complex educational and development intervention to improve palliative care in nursing homes. Little research has investigated processes in the cross-cultural adaptation and implementation of interventions in palliative care across countries, taking account of differences in health and social care systems, legal and regulatory policies, and cultural norms. This paper describes a framework for the cross-cultural development and support necessary to implement such an intervention, taking the PACE Steps to Success programme as an exemplar.

**Methods:**

The PACE Steps to Success programme was implemented as part of the PACE cluster randomised control trial in seven European countries. A three stage approach was used, a) preparation of resources; b) training in the intervention using a train-the-trainers model; and c) cascading support throughout the implementation. All stages were underpinned by cross-cultural adaptation, including recognising legal and cultural norms, sensitivities and languages. This paper draws upon collated evidence from minutes of international meetings, evaluations of training delivered, interviews with those delivering the intervention in nursing homes and providing and/or receiving support.

**Results:**

Seventy eight nursing homes participated in the trial, with half randomized to receive the intervention, 3638 nurses/care assistants were identified at baseline. In each country, 1–3 trainers were selected (total *n* = 16) to deliver the intervention. A framework was used to guide the cross-cultural adaptation and implementation. Adaptation of three English training resources for different groups of staff consisted of simplification of content, identification of validated implementation tools, a review in 2 nursing homes in each country, and translation into local languages. The same training was provided to all country trainers who cascaded it into intervention nursing homes in local languages, and facilitated it via in-house PACE coordinators. Support was cascaded from country trainers to staff implementing the intervention.

**Conclusions:**

There is little guidance on how to adapt complex interventions developed in one country and language to international contexts. This framework for cross-cultural adaptation and implementation of a complex educational and development intervention may be useful to others seeking to transfer quality improvement initiatives in other contexts.

## Background

There is evidence that palliative care benefits people with life limiting conditions at all ages and has been endorsed by the World Health Assembly [1]. Palliative care seeks to improve quality of life, by addressing physicial, psychosocial and spiritual needs, and is regarded as a complex intervention best delivered by a multidisciplinary team [2, 3]. The Lancet Commission has highlighted the globally inadequate current provision of palliative care and pain relief, especially for older people [4].

Nursing homes, defined here as care homes or long term care facilities with on-site nurses, are an important place of care for a proportion of older people with high levels of physical, psychological and or social need, including those with dementia [5, 6]. Nursing home staff may have limited care qualifications, low salaries and high turnover [7, 8]. To address the needs of residents who die in these settings, palliative care interventions have been developed to support staff to deliver care for dying residents such as the Gold Standards Framework for care homes [8] and the ‘Route to Success’ programme [9, 10]. Studies suggest that such interventions increase staff knowledge and confidence to care for older residents and their families, as well as decrease the proportion of residents transferred to hospital to die [11–13]. All interventions are initially developed within specific cultural, legal and linguistic contexts. Failure to adequately test novel interventions and ensure their safe and effective implementation may result in inadequate training and insensitive care as illustrated by the Liverpool Care Pathway, a British end of life care intervention that was withdrawn following public protests [14].

Kitson et al. [15] acknowledge the complexity of introducing change in practice and argue it is a balance between the *evidence* incorporated within the new programme, the *context* into which it is being implemented and the degree of *facilitation* required [16–18]. The Promoting Action on Research Implementation in Health Services (PARiHS) framework suggests that there is a continuum from ‘low to high’ ‘weak to strong’ in relation to the level of the evidence, context and facilitation [19, 20]. A high facilitation model has been shown to be beneficial when implementing a complex palliative care intervention into nursing homes [12, 21].

There is little guidance on cross-cultural adaptation of palliative care interventions, where the potential sensitivities and cultural aspects of dying are acknowledged. For example, disclosure of prognosis varies widely across Europe, as do legal and ethical regulations, such as advance directives or medication availability. This paper offers a framework for the cross-cultural development and support necessary to implement a complex palliative care intervention in nursing homes. The word *framework* describes the structure outlining the process undertaken, with the PACE Steps to Success programme used as an exemplar [22, 23].

## Methods

The PACE Steps to Success programme was implemented as part of the PACE cluster randomised control trial across seven European countries to improve palliative care in nursing homes [23]. It was informed by the PARiHS framework [15].

The training programme and materials were prepared in English with the content being compiled from evidence of previous palliative care programmes in the UK. Materials were then translated to the langauges of participating countries. Two training events in the UK were delivered to 16 country trainers from the seven countries. A train-the-trainers model was adopted with the aim that training in the intervention and in specific teaching methods and styles would be cascaded from: international trainers to country trainers, country trainers to PACE coordinators, PACE coordinators to nursing home staff with all receiving considerable facilitation and support [15].

We draw upon collated evidence from minutes of three international consortium meetings, qualitative evaluation of training delivered through 34 group interviews with care staff delivering the intervention in nursing homes (a total of 151 staff), 25 group interviews with PACE coordinators (total of 73 PACE coordinators), 29 interviews with nursing home managers and an on-line group interview with 16 country trainers [24].

The cross-cultural development and implementation of the PACE Steps to Success programme followed three phases:

- Phase 1: adaptation and preparation of resources;
- Phase 2: training and implementation using a train-the trainer model;
- Phase 3: supporting the implementation.

### Phase 1: adaptation and preparation of resources

Complex interventions require careful description of underpinning evidence, procedures and processes to be followed prior to their implementation to ensure fidelity. In the PACE trial, this involved the development and cross-cultural adaptation of resources, written documents and tools (structured measures). All documents were initially written in English.

The PACE Steps to Success Programme drew upon the ‘Route to Success’ programme [10] orginially developed for England and Wales [25]. The tools were selected because they were appropriate for use within nursing homes, including residents with dementia, and this organisational setting [23].

**Fig. 1:**
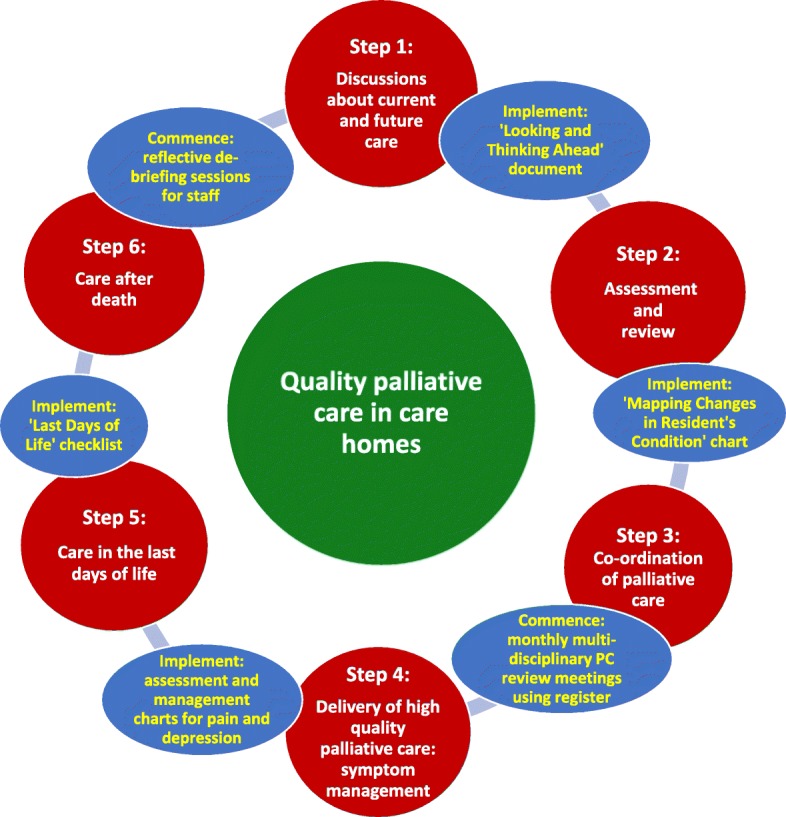
A diagram of the PACE Steps to Success programme and their corresponding tools

The PACE Steps to Success comprised six sequential steps, namely: discussions about current and future care; assessment and review; monthly multi-disciplinary palliative care review meetings; delivery of high quality palliative care (focused on symptoms of pain and depression); care in the last days of life; and care after death (Fig. 1) [23].

### Phase 2: training and implementation of the programme using the train-the-trainer model

There is evidence that the train-the-trainers model embeds knowledge better than didactic education since those cascading knowledge listen more comprehensively to the training because they have to teach it to others [26]. It enhances sustainability of changes occurring in practice. In the PACE trial, implementation of the PACE Steps to Success programme used a train-the-trainers model that was cascaded through:

- international experts - in English
- within country trainers who visited each intervention arm nursing home every 10 to 14 days during the 12-month implementation period and were responsible for training the PACE coordinators based in each nursing home – in the local language.
- PACE coordinators who were designated staff employed within the nursing homes, supported the sustainment of the implementation within the setting.

**Fig. 2:**
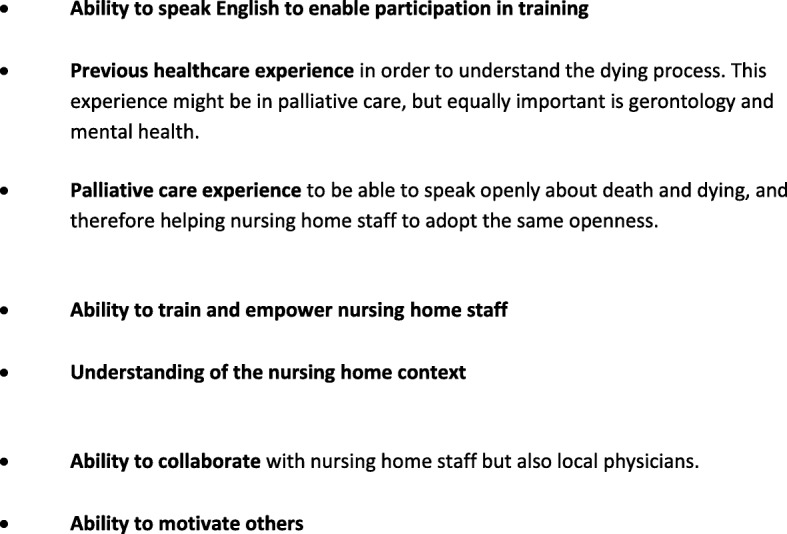
Criteria for appointment of the Country Trainer

To ensure equity between countries, the country trainers were appointed by research partners using specific criteria (see Fig. 2), which indicated the skills and expertise, required.

### Phase 3: supporting the implementation

There is evidence that training alone may not be sufficient to embed changes in practice [27]. Therefore, we designed an implementation process that included cascading a high level of support and facilitation of the programme [28], informed by the PARiHS framework [15]. The nursing home context was perceived as ‘low’ in relation to its knowledge and practice of palliative care so required ‘high’ evidence and ‘high’ facilitation [15]. Examples of high level support and facilitation included: monthly internet-based international groups for country trainers and mentorship from national research leaders. Country trainers then supported the nursing home PACE coordinators by visiting each nursing home every 7–10 days.

## Results

**Fig. 3:**
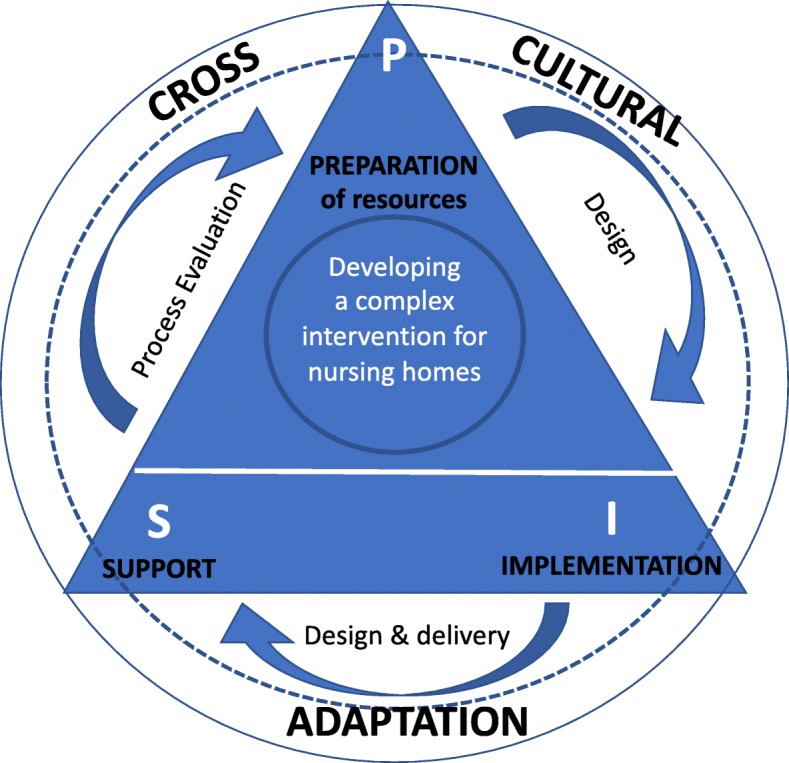
A cross-cultural adaptation framework

In this section, we describe the cross-cultural adaptation processes in relation to the implementation of a complex intervention, highlighting them in the context of the three phases. The cross-cultural adaptation of the programme was regarded as permeating all aspects of the development and implementation of the programme (see Fig. 3). Results of the PACE cluster randomised control trial [29] and a formal evaluation of the PACE trial implementation process are reported elsewhere [24].

In total 78 nursing homes participated in the PACE trial, with half randomized to receive the intervention [29]. In each nursing home, one or more managers participated, and 3638 nurses and care assistants were identified at the start of the trial across all nursing homes. In each country, 1–3 trainers were selected (total *n* = 16) to deliver the intervention. The country trainers had diverse professional backgrounds including seven nurses, four physicians, three psychologists, one social worker and one sociologist. All country trainers were employed part-time, and most combined this with other roles. We report processes of implementation in relation to the three-phase framework used to guide the cross-cultural adaptation and implementation.

### Adaptation and preparation of resources

**Fig. 4:**
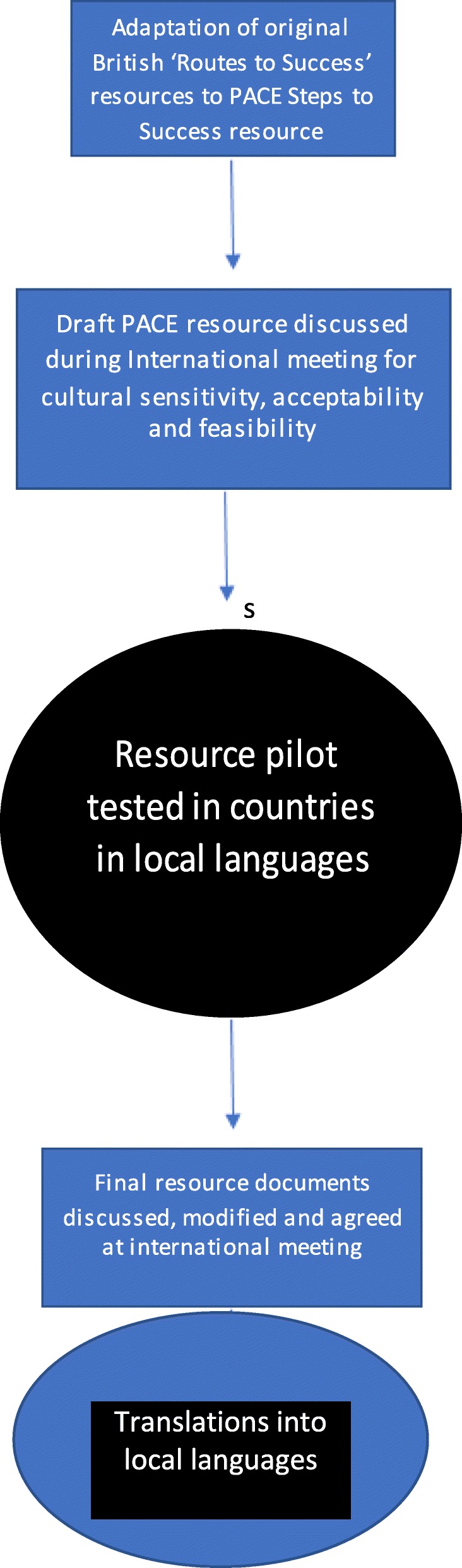
Flow chart – cross-cultural adaptation of resources

The core document was discussed at an international team meeting, which included partners who worked with older people and people with dementia; the content of the document was reviewed and revised for cultural sensitivity and clinical appropriateness. The document was then reviewed by staff for acceptability, feasibility and cultural appropriateness in two nursing homes in each country who were not included in the trial. Two further documents were developed that built upon the core document. One for country trainers that included a detailed explanation of all training sessions to support the PACE coordinators to implement the programme (see Fig. 1). Another version for PACE coordinators had less information than the country trainers but more information than the core document. All documents were translated from English into trial country languages (see Fig. 4).

Preparation of resources was an iterative process. During meetings with the international project partners, it was agreed to simplify the content, focus on specific measurable outcomes and to introduce generic tools where these were not already in use in the nursing homes such as structured pain assessment forms. These discussions highlighted different cultural norms such as diagnostic disclosure practices, which influenced the acceptability of advance care planning (ACP) (Step 1). For example, while ACP was well understood in Belgium, the Netherlands, Switzerland and UK, it was not widely recognised in Finland, Italy and Poland [24]. There was considerable discussion regarding country differences in legal recognition of cardiac resuscitation orders and they were removed from the final document. Likewise, following much debate, assessment of specific symptoms was confined to pain and depression, rather than other generic symptom assessment. In Poland, for example, there was a greater hierarchical medical model compared to the UK; this meant that assessment of symptoms relied on medical staff rather than the nursing team as often is the situation in UK nursing homes. We also recognised differing national legislation and policies that for example influenced prescribing of opioids [30]; these were not available in nursing homes in Poland.

### Training and implementation

**Fig. 5:**
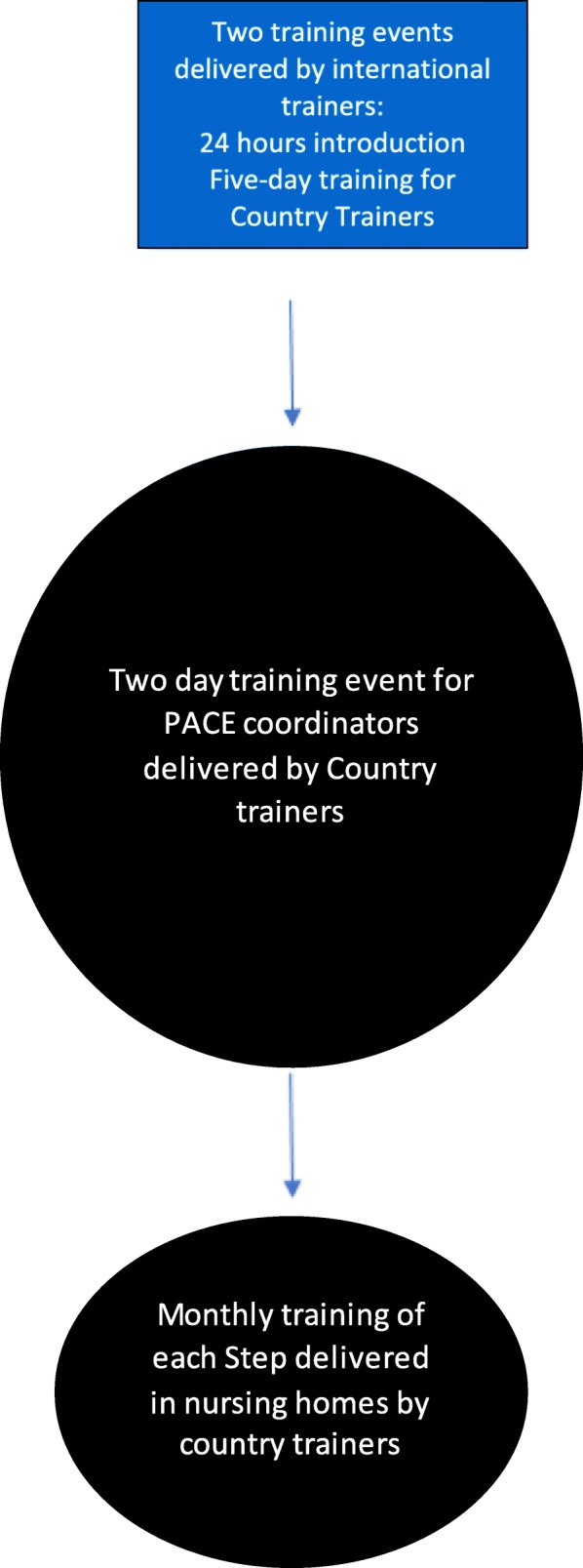
Flow chart to show training

The train-the-trainers model was applied by bringing all country trainers together for a one-day meeting to introduce the PACE Steps to Success programme and to meet their peers. This was followed by an intensive five-day training event three months later that involved outlining the PARiHS model [15], role modelling teaching styles, and, practising the training activities that country trainers would deliver in their own countries during the preliminary phase of the implementation to PACE coordinators based in nursing homes (see Fig. 5).

The five-day training gave country trainers the opportunity to engage more fully with the PACE intervention and the resources, and to understand cultural differences in how general palliative and end of life care was managed in nursing homes in participating countries. Both these training events were conducted in English by two international trainers (JH, KF). During the training and afterwards, country trainers returned to their country and discussed with clinical colleagues the cultural differences in legal and ethical aspects of palliative care. Where necessary, further changes were made to the individual country resources.

Following the five-day training, country trainers organised two days of training for the PACE coordinators from the intervention arm nursing homes. This training was delivered in the local language and brought together all the PACE coordinators prior to the implementation of the PACE Steps to Success programme. PACE coordinators were qualified nurses or senior care assistants and normally there were two or three identified per nursing home, to allow for flexibility in working shifts. They helped to organise the monthly within nursing home training on the six steps being delivered by the country trainers. In larger nursing homes, the training was sometimes repeated as much as three times during the month, to ensure access for all staff. The training was for nursing home staff, medical staff and any other staff external to the nursing home who were involved in palliative care.

A number of cultural challenges became apparent; for example, hierarchical relationships between different professional disciplines which influenced communication and decision making towards the end of life. In countries such as Poland and Italy, nurses appeared to have a lower social status than physicians, so although they had more opportunities for interaction with family members of dying residents, more salience was afforded to medical communication about end of life decision making [31]. There were differences too in the educational level of those providing care to residents and how that care was documented, in paper or online records. In some countries, nursing homes used electronic care planning which included pain assessment scales which were different to those recommended in the PACE resources.

### Supporting the implementation

**Fig. 6:**
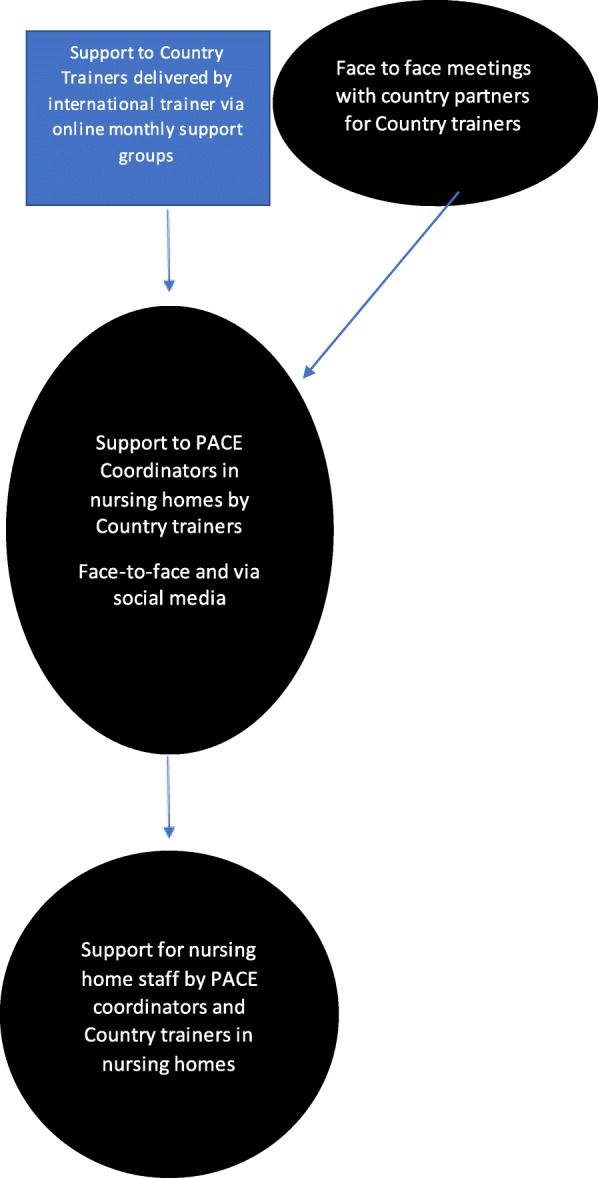
Flow chart – support for implementation

Support was cascaded down and up through each level. Country trainers were supported by an international trainer in English via online groups, and secondly, by lead research partners in local languages. Country trainers in turn supported PACE coordinators within the nursing homes who assisted staff in implementing the intervention (see Fig. 6).

The online groups for the country trainers were facilitated in an action learning style whereby the international trainer used open questions about experiences and challenges encountered in implementing the programme, which also facilitated peer learning [32]. Once the groups became established, prearranged monthly on-line meetings were arranged. However, even with prior notice, the unpredictable nature of work meant that some country trainers were not able to attend these meetings due to demands from their other roles, personal commitments or illness. Another key issue for the country trainers attending the on-line support meeting was that the majority were not speaking in their first language.

Country trainers were regarded as key people in delivering the PACE intervention, but most had relatively isolated roles. Additional monthly support was given by the research leads. These meetings were designed to deal with predominantly practical and logistical concerns, but also addressed the emotional labour of the sensitive topics raised by the PACE Steps to Success programme.

A further dimension of support that needed to be integrated into the implementation process concerned the palliative care focus of the intervention. In some countries, explicitly talking about dying with the residents, rather than relatives, and making plans for the future such as ACP, was not culturally acceptable. For example in Italy and Poland, end of life communication and decision making was normally conducted with family members rather than residents [31] which meant that Step 1 was poorly implemented.

The role-modelling support by country trainers to PACE coordinators was regarded as showing the importance of facilitation and helped with organisational and practical changes. PACE coordinators were at the forefront of addressing barriers to change, and used their pre-existing relationships with nursing home staff, managers and external professional staff. Supporting staff in the nursing homes to address culturally challenging issues created demands both for the country trainers and the PACE coordinators. For example, in the UK where most nursing homes are independent ‘for-profit’ organisations, the country trainer became aware that nursing home staff often lacked access to evidence-based resources and mutual support, so she established a ‘Facebook’ group which successfully overcame their perceived isolation. It also facilitated exchange of resource material. The leadership ability and continuity within the nursing home was also of particular importance. The support of the PACE programme by the nursing home manager was critical for a good outcome.

## Discussion

This paper highlights the importance of a structured framework for cross-cultural adaptation and implementation of a complex educational and development intervention (which has been designed for one country and language) when transferring it to international contexts. By coordination and integration of the three phases, namely, adaptation and preparation of resources, delivering the intervention within a train-the-trainers model, and cascading support throughout those facilitating the intervention, we anticipate that this framework may have a resonance and applicability beyond the PACE project. The PACE project was largely implemented in well resourced countries, so how such a framework is used in low-middle resourced countries needs further research.

### Summary of the implementation process of the PACE steps to success programme

This account of the design, adaptation and execution of a complex palliative care intervention for nursing homes provides evidence of the important methodological and practical issues that arise in implementing interventions for residents near the end of life. Using the three phases, we successfully adapted a British programme for use in European nursing homes. The final version of the PACE Steps to Success Programme Information Pack and tools are now freely available in Dutch, English, Flemmish, Finnish, Italian and Polish (French forthcoming) [33]. As anticipated we faced a number of challenges during implementation related to the different stages of development of palliative care and its integration into the nursing home context [6], and hierarchical organisational structures in nursing homes, and unexpected barriers such as their apparent isolation from other similar organisations. Overall, implementation quality was variable, with some difference in staff attendance at training between countries and facilities as described more fully in the process evaluation [24].

Adoption of evidence-based guidelines into practice present some difficulties within the nursing home context because of low levels of staff qualifications, high staff turnover and limited investment in education [34]. However, there is evidence that nursing homes are keen to embrace palliative care [21, 35] despite the considerable barriers that are often present [36]. Both a bottom up and a top down approach to change is important within the often hierarchical structure of nursing home organisations. The dynamic sustainability framework highlights that there needs to be a ‘fit’ between an intervention and the context in order to optimize benefit [37]. However, there was limited opportunity for flexibility within the PACE Steps to Success programme because it was implemented within the context of a cluster randomised control trial [23].

The operationalisation of complex interventions into core elements with clear guidance on implementation processes are recommended. The preparation of resource materials needs to achieve a balance between detailed instructions, length of documents and ease of reading for busy clinicians. In the context of the PACE trial, we adapted three documents for the PACE Steps to Success programme, drawing upon an initial English version. Whilst the documents were always available to be referred to, and a constant reminder of the nursing home’s involvement in the intervention, they were often kept in the manager’s office with little evidence that care workers and nursing staff used them. Availability of online resources might provide better options for nursing homes with technologically advanced health care systems.

The train-the-trainers model was a means of cascading knowledge and skills from the UK international trainers, to country trainers within the seven European countries, to PACE Coordinators within each nursing home, and, then to nurses and other workers caring for residents with palliative care needs on a daily basis in nursing homes. When comparing this to a similar large study in the USA [26], the PACE study was arguably more challenging because of the different languages and cultures throughout Europe. There were also organisational cultural differences across nursing homes partly due to the differing funding models [6]. For organisational culture to change, it requires the leadership to be willing to adapt to new ways of doing things [38].

Within the PACE project, considerable emphasis was put on the facilitation and support process from the beginning. Previous work in the UK on complex palliative care interventions in nursing homes had found the PARiHS framework useful to guide the implementation process [11, 20]. The PARiHS framework takes account of the type and nature of the evidence being introduced, the social and organisational context and the elements that facilitate the implementation – if either the evidence or the organisational context are ‘weak’ then the facilitation within the intervention needs to be ‘strong’ [15]. A cluster randomised control trial implementing a palliative care programme found a significant association between the intensity of external facilitation and nursing homes completing the implementation through to accreditation [21, 39]. Those nursing homes that received both high facilitation and action learning (where the manager were involved in monthly action learning sets) had significantly better results. It appears that the individual nursing home culture plays a considerable part in it’s readiness for change and willingness to accept the facilitation and support being offered, as highlighted in previous research [40, 41].

Recent evidence from the PARiHS group in their international randomised controlled trial in long term care facilities examines in more depth the role of facilitation when bringing about change [41]. The study highlights the significance of organisations in prioritising commitment to change. Whilst the PACE Coordinators were willing to participate and were appointed by the nursing home managers, sometimes the authority given to them was not sufficient to implement changes in practice. PACE Coordinators needed to be able to be internal facilitators of change, and the skills required to do this were not always available within the nursing homes. Further work is required to understand the different impacts of external and internal facilitation upon the implementation processes within nursing homes.

### Strengths and limitations

The strength of the framework is the integration of three levels of cross-cultural development within the implementation. We were fortunate to draw upon international experts and international advocacy organisations such as Alzheimer’s Europe, European Association for Palliative Care and Age Platform, including the input of people with dementia, to assist in the cross-cultural adaptation processes. This variety of input from seven countries increases the applicability of the framework to other settings. However, the methods used in developing the framework do not enable us to verify our findings, except by presenting the PACE Steps to Success as an exemplar. Our implementation activities were shaped, and to some extent constrained, because the PACE Steps to Success intervention was tested within a cluster randomised controlled trial. Therefore, it did not allow for a lot of flexibility in implementation. The framework remains to be tested in implementing other interventions.

A limitation of our study is the difficulty of contextualising a structured intervention in an institutional environment that continually shifts and transforms, as resident’s needs, and staff, change. A further limitation in the study was the dominance of the English language and the cultural assumptions that underpinned the initial programme and training. For example, the difficulty in translating English metaphors or idioms became readily apparent when translations were made.

The PACE Steps to Success programme was initially drafted without input from those involved in the day-to-day care of residents in nursing homes in the seven countries. As a result there was a lack of awareness of a number of things. The cultural sensitivities and practicalities for those working in countries where general palliative care was largely unknown outside oncology settings. Further still, whilst the PACE programme and tools were considered very important, they were paper-based; at the time the study was being designed, few UK nursing homes had electronic care planning systems. This was in contrast to many nursing homes within the majority of PACE countries who already had electronic records in place within their nursing homes. As the PACE intervention used paper copy tools, there was tension between the two systems and potential redundancy of effort. It is important in future that tools are integrated into the nursing home record system so that staff can use them more effectively.

It is important to consider within this study whether the intensity of the programme itself was in fact ambitious within the limited period of of the trial. Not only was the programme attempting to implement a new palliative care organisational structure into each nursing home but it was also expecting staff to introduce new clinical tools. It could be argued that whilst both are important, a less linear approach to implementation and instead a two-stepped approach where one establishes the organisational structure first might have been better. In a final international meeting this was discussed with the importance of facilitating greater exterrnal multi-disciplinary support (such as monthly palliative care review meetings) and staff support following a resident’s death was seen as important starting points across cultures.

## Conclusions

In conclusion, the framework for cross-cultural adaptation and implementation of a complex intervention may be useful to others seeking to transfer quality improvement initiatives in other contexts. The PACE Steps to Success intervention has provided an opportunity to look in depth at the process of cross-cultural adaptation of the intervention to improve palliative care in nursing homes. Three elements, namely: adapting and harmonising the resources, use of a train-the-trainers model, and cascading the support throughout the implementation period, have been identified as a possible framework with a focus on the importance of cross-cultural adapation within each element.

The framework requires further testing. More attention needs to be given during the design of the tools to the views of service users, nursing home staff and managers about the usefulness of the care model, opportunities and barriers within the context, and culturally sensitive outcomes.

## Data Availability

All data are archived at the Division of Health Research, Lancaster University, United Kingdom and at the relevant consortium universities and may be obtained from the corresponding author. As this paper concerns process and implementation, we have not placed the dataset in a public repository.

## Abbreviations

JH: Jo Hockley
KF: Katherine Froggatt
PACE: Palliative Care for Older People (name of the project)
PARiHS: Promoting Action on Research Implementation in Health Services
UK: United Kingdom
USA: United States of America
